# Empowering Pacific Patients on the Weight Loss Surgery Pathway: A Co-designed Evaluation Study

**DOI:** 10.1007/s11695-024-07084-w

**Published:** 2024-02-12

**Authors:** Tamasin Ariana Taylor, Grant Beban, Elaine Yi, Michael Veukiso, Genevieve Sang-Yum, Ofa Dewes, Wendy Wrapson, Nalei Taufa, Andrew R. T. Campbell, Richard J. Siegert, Peter Shepherd

**Affiliations:** 1 Department of Molecular Medicine and Pathology, Faculty of Medical and Health Sciences, The University of Auckland, Building 507, Room 1090, 22-30 Park Avenue, Grafton, Auckland, 1023 New Zealand; 2 General Surgery Department, Auckland City Hospital, Te Whatu Ora, Te Toka Tumai, 2 Park Road, Grafton, Auckland, 1023 New Zealand; 3 School of Social Work, College of Health, Massey University, Albany, North Shore, Auckland, 0745 New Zealand; 4 Centre of Methods and Policy Application in the Social Sciences, The Faculty of Arts, University of Auckland, 12 Grafton Road, Auckland, 1010 New Zealand; 5 Langimalie Research Centre, Tongan Health Society, M20 Business Park, 86F Plunket Avenue, Manukau, Auckland, 2104 New Zealand; 6 Faculty of Health and Environmental Sciences, School of Clinical Sciences, Auckland University of Technology, 90 Akoranga Drive, Auckland, 0627 New Zealand; 7 Department of Anthropology, The Faculty of Arts, The University of Auckland, 22 Symonds Street, Auckland, 1010 New Zealand

**Keywords:** Pacific-led, Co-design, Bariatric surgery, Weight loss surgery, Metabolic surgery, Preoperative surgery work-up, Culturally competent, Pacific cultural competency

## Abstract

**Purpose:**

Despite having the highest medical needs by population for weight loss treatment, Pacific patients in Aotearoa New Zealand face substantial levels of attrition in publicly funded weight loss surgery programs. In collaboration with the Auckland City Hospital bariatric surgery team, a Pacific-led preoperative weight loss surgery program was co-designed, delivered, and evaluated between 2020 and 2023.

**Materials and Methods:**

This was a single-arm, prospective co-designed evaluation study that took place at Auckland City Hospital in Aotearoa New Zealand. Participants were Pacific patients (*n* = 14) referred to the weight loss surgery program. Survey and video diaries were analyzed to determine if the program had the potential to increase Pacific patient retention through the preoperative stage of weight loss surgery, increase surgery completion rates, and improve the quality of treatment experiences.

**Results:**

Nine out of 14 participants attended all preoperative sessions. Six participants subsequently underwent weight loss surgery. Program components that had positive impacts on patient success and satisfaction were accessibility, information quality, having Pacific role models, cultural safety, and the group support system. The patients found the program to be culturally anchored and there was support for the implementation of the program going forward.

**Conclusion:**

This study demonstrated how a culturally anchored intervention can increase patient retention for those patients who may not respond to mainstream treatment. Adjusting existing preoperative weight loss surgery programs to integrate Pacific-led models of healthcare has the potential to increase Pacific patient resiliency to follow through with surgery.

**Graphical Abstract:**

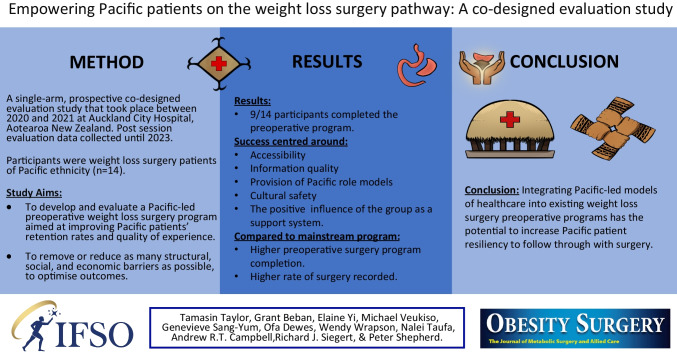

## Introduction

Weight loss surgery is one of the most effective long-term treatments for obesity-related co-morbidities [1–3], outperforming lifestyle treatments (e.g., diet and exercise) [4] and anti-obesity medications [5]. A reported 84% of patients with type 2 diabetes experience complete remission after weight loss surgery, and conditions such as sleep apnoea, hyperlipidaemia, and hypertension are resolved or significantly improved [6, 7].

Pacific populations in Aotearoa New Zealand (NZ), have the highest prevalence of obesity and obesity-related co-morbidities of all ethnic groups. They are 2.3 times more likely to be obese, three times more likely to have diabetes, and four times more likely to have kidney failure than non-Pacific people [8]. Despite having the greatest need for obesity treatment, Pacific populations have the lowest engagement and completion rates for publicly funded weight loss surgery. The number of eligible Pacific patients receiving publicly funded weight loss surgery in NZ shows a substantial disparity, with 0.7 surgeries undertaken per 1000 for Pacific ethnicities, compared with 3.0 for NZ Europeans [9]. A later study calculated that eligible Pacific people received approximately half the rate of publicly funded surgeries as European and Māori each year over the 2013–2017 period [10].

Once referred to a weight loss surgery program, Pacific patients moving through the preoperative stage are highly vulnerable to attrition. During the preoperative stage of surgery, patients must attend specialist appointments (e.g., with the surgeon or dietitian) and group support sessions normally located within the hospital. Patients need to demonstrate eating and exercise patterns relevant to preparing for the upcoming surgery. Lifestyle improvements facilitate more effective treatment outcomes through higher post-surgical weight loss, nutritional management, and long-term weight loss maintenance. If a patient cannot attend all appointments and group education sessions and demonstrate they have made sufficient lifestyle changes before surgery, they are discharged from the pre-surgery program and referred to their GP or specialist. In one major program located at Auckland City Hospital (NZ) between 2007 and 2016, 73% of Pacific patients referred for weight loss surgery dropped out before surgery took place, and for Pacific males, the attrition rate was 87% [11]. Another study at Counties Manukau, Auckland, NZ, found that only 28% of Pacific patients underwent surgery after being accepted into the surgery program compared to 63% of NZ European patients [12].

The authors’ previous work has identified the challenges facing patients of Pacific ethnicity at the preoperative surgery stage. Twenty-one health sector professionals who worked directly with Pacific patients (surgeons, dietitians, nurses, GPs, psychologists) or in wrap-around services (community health workers, public health physicians) were interviewed to explore factors contributing to Pacific patients’ disproportionately high preoperative attrition rates [13]. Following this, 15 former weight loss surgery patients of Pacific ethnicity who had entered weight loss surgery programs at two hospitals in the wider Auckland region were interviewed [14]. The themes arising from these interviews were used to develop a Pacific-led preoperative weight loss surgery program by targeting the barriers to program completion (see Table 1 for changes to the mainstream program).

**Table 1:** Details of the differences between the mainstream preoperative program and the Pacific-led program

Standard support program	Pacific-led support program	Purpose of change
Patient recruitment to the program		
Patients identified as eligible by the ACH bariatric surgery team are sent a letter inviting them to attend an information session held at the hospital, usually at 2 pm on a weekday If patients decide to participate in the weight loss surgery program after attending that information session, they begin the standard group support sessions	The ACH bariatric team identifies eligible patients of Pacific descent and seeks their consent for the project team to contact them. If consent is granted, the principal investigator (PI) phones the patients and explains the purpose of the Pacific-led support program. Patients are invited to attend the initial information session, which is held on a Saturday afternoon, followed by the other Pacific-led group support sessions over the following months	By phoning patients individually, and introducing herself and the program, the PI aims to establish a positive and trusting relationship with the patient at the outset of the program Together the patient and PI can work through potential barriers, such as transport, session times, and social support needs
Location and timing of sessions		
Group support sessions are held in a clinical centre during normal working hours	Group support sessions are held in a community centre in a private room on a Saturday afternoon	This aims to reduce barriers associated with fear of hospitals, shyness about needing weight loss surgery, and difficulties around parking and transport
Arrangement and conduct of sessions		
Patients sit in rows in a conference room. The surgeon or other bariatric team member talks to the group about what the surgery involves and the changes patients need to make. Questions are invited at the end	Sessions follow a Pacific protocol with: • Seats arranged in a semi-circle • Opening and closing prayers • Introductions around the group • Presentation by a health professional (e.g., surgeon or bariatric nurse) • *Talanoa* and closing remarks • Light refreshments	This aims to create a culturally safe environment and to build trust and mutual respect between the health professionals and patients
Pacific role models		
Role models are not currently employed in the standard support program. However, patients can contact the bariatric nurse by mobile phone if they have any concerns	The various Pacific weight loss surgery role models attend all sessions alongside the health professional and facilitator. In the first session, two role models (one female, one male) relate their weight loss surgery journey. A video clip of one of the role models’ journey is played. Patients can ask the role model questions during sessions or at a later date via SMS, telephone, or email	The need for role models of Pacific ethnicity was a key improvement to the standard program suggested by Pacific patients interviewed in the PI’s previous interview study Being able to talk about the surgery journey with others who have successfully completed surgery helps build patients’ confidence and overcome their fears and concerns. Emphasizing the benefits of surgery motivates them to stay in the program
Worldview of health and illness		
Sessions focus on the western biomedical model of health, whereby surgery is a means of improving physical health and avoiding an early onset of death. Surgery preparation focuses on improving diet and increasing exercise	Sessions reflect a holistic Pacific worldview of health, which includes physical, mental, social, and spiritual wellbeing [15]. Surgery preparation focuses on improving diet and increasing exercise but also on developing skills to cope with stress and difficult social situations in positive ways	By moving away from a Eurocentric, individualistic model of health towards a more holistic Pacific model incorporating the need to be a productive member of one’s family and community, patients’ motivation to go through with surgery is enhanced
Frequency of sessions		
After the initial information session, patients attend three group education sessions, one each run by the clinical nurse specialist, dietitian, and health psychologist	After the initial information session, patients attend four group support sessions, one each run by the clinical nurse specialist and health psychologist and two run by the dietitians	The higher number of sessions aims to improve connection with the program and increase the level of support provided to patients
Session facilitation		
Group education/support sessions are run by mainly NZ European bariatric surgery team members (nurse, dietitian, health psychologist)	Group education/support sessions are facilitated by the PI, who is of Pacific heritage. She is assisted by up to two Pacific patient role models per session. It is made clear to everyone at the outset that meetings are confidential and individual information is not to be discussed outside of the meetings	This aims to increase cultural safety, to encourage patients to feel comfortable and less concerned about being judged. It is emphasized that everyone is part of each other’s weight loss surgery support group or *Aiga* (family) and that participants are all on a journey together
Family involvement		
Support people are allowed to attend group support sessions	Family members are encouraged to attend and are involved in discussions. Children can watch a movie and play in an adjoining room. Babies and toddlers can remain with their parents	Encouraging families to attend provides practical support and reduces social stigma. Patients know that, if they choose to, they can bring their family with them on their journey
Practical barriers		
Patients pay for parking and travel. Sessions are held during normal working hours	Practical barriers are reduced according to each individual’s needs. Parking and petrol costs are reimbursed for those who need them, and sessions are held outside normal working hours	These initiatives help to reduce practical barriers that patients may face

## Study Aims

The primary aims of the current study were:

- To develop and evaluate a Pacific-led preoperative weight loss surgery program based on Pacific health values to improve Pacific patients’ retention rates and quality of experience.
- To optimize outcomes and remove or reduce as many structural, social, and economic barriers as possible.

The focus was on the preoperative stage because this was identified as the point at which Pacific patients are disproportionately vulnerable to disengaging with the program and dropping out. Specific requirements included reducing the time spent in clinical spaces and increasing access to culturally safe spaces when providing support services; minimizing the financial burden arising from childcare, transport, food, and other costs; and employing Pacific role models to support patients and their families.

The secondary aims of this study were:

- To determine whether the Pacific-led preoperative weight loss surgery program had the potential to increase retention for Pacific patients.
- To identify which components of the program positively impacted patient satisfaction.

## Method

This single-arm, prospective evaluation study employed quantitative and qualitative methods to analyse survey and video diaries data. The study took place in Auckland, Aotearoa New Zealand, and was limited to patients within the catchment area of the Auckland District Health Board (ADHB) (now part of Te Whatu Ora) who were referred to the Auckland City Hospital (ACH) weight loss surgery program. The study was supported by the ACH bariatric team, who co-designed the program, made initial contact with patients to recruit them into the study, and conducted the preoperative education sessions. The program was also co-designed with two Pacific role models who had previously undergone surgery. From a medical point of view, there was no difference between the two programs: the differences lay in the support structures and services provided.

Differences to the program included the following: the first information and subsequent group education sessions were held in a community space rather than at a clinical centre, and sessions were led by the bariatric team members accompanied by a Pacific facilitator and one or two Pacific role models who had gone through the surgery process previously. The study covered travel and parking costs if participants needed this assistance, and patients were welcome to bring along family members. A key session component was group *talanoa*, a Pacific form of interaction that “…allows people to engage in social conversation which may lead to critical discussions or knowledge creation that allows rich contextual and inter-related information to surface…” [16]: 2009, p. 24). This enabled in-depth conversation around the surgery process. In addition to the medical preparation for surgery, each session involved discussing some of the stigma, barriers, and enablers of the patients’ surgery process.

The initial information session occurred on 31 October 2020, and the group support sessions ran from 22 May to 10 July 2021. The inclusion criteria were being of Pacific heritage, 18 years or older, and acceptance into the ACH weight loss surgery program. After attending all five sessions, participants completed a post-preoperative session survey. This was designed to measure the extent to which participants found these sessions useful in their preparation for weight loss surgery and to gather qualitative data about their experiences. Participants then followed the normal clinical path to surgery.

## Results

Of 20–25 eligible patients invited to the Pacific sessions, 15 expressed an interest in attending the information session, and 12 turned up on the day. Another two subsequently attended a second scaled-down session, resulting in 14 attendees. Nine out of 14 participants attended five education/support sessions, as was required to remain eligible for surgery. Six participants missed one of the community sessions and had to attend one mainstream session to make up for the missed session. Four attended a mainstream health psychology session, and two attended a mainstream dietitian session. Of the 14 participants, seven were born in NZ, and seven were Island-born. The average age was 45 years; 11 identified as female, two as male, and one as transgender. Of the 14 participants, nine identified as having Samoan heritage, four as Cook Island Māori, one as Tongan, one as Niuean, and one as having Tokelauan heritage. The average weight was 148.8 kg, and the average BMI was 53 kg/m^2^ (range 42.4–72.6 kg/m^2^). All participants had one or more co-morbidities, the five most prevalent being obstructive sleep apnoea (*n* = 10), type 2 diabetes (*n* = 9), hypertension (*n* = 7), joint/back pain (*n* = 5), and dyslipidaemia (*n* = 4).

Nine out of 14 participants attended all preoperative support sessions. Reasons for five patients dropping out before completion of these sessions included limited understanding of English, mental health issues, the need to look after family members, death of the patient, and no reason given. Six of the nine remaining participants underwent weight loss surgery, two dropped out after the sessions concluded, and one was still in the program. Reasons for dropping out or not proceeding to surgery after the education sessions included change of mind, not attending further pre-surgery appointments, and failure to meet preoperative medical requirements. The retention rate for surgery was 43% (6/14).

**Fig. 1:**
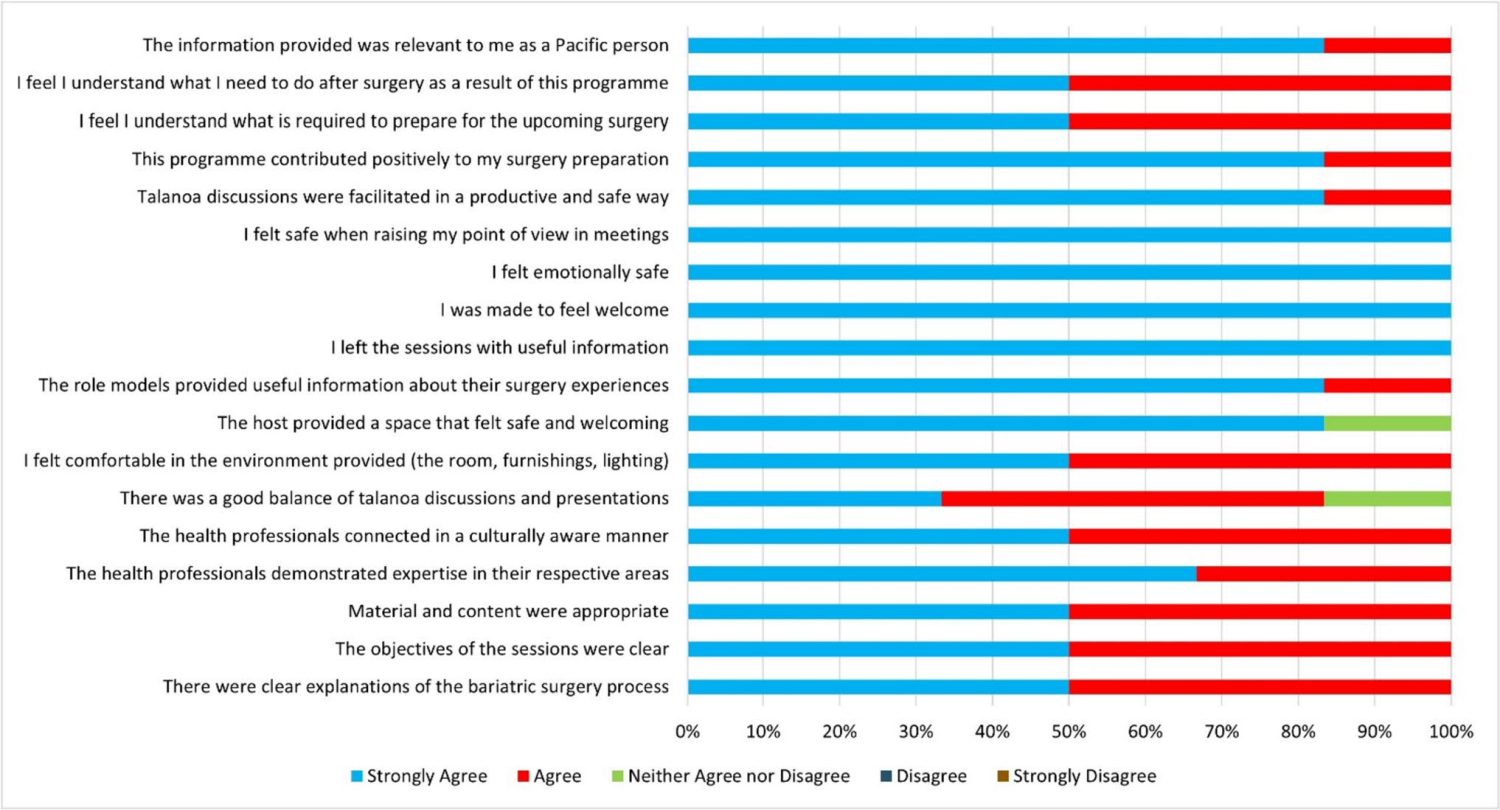
Participant feedback on the content of preoperative information sessions

Data from the post-preoperative session survey indicated that components of the program that had a positive impact on patient success and satisfaction centred around accessibility, information quality, having Pacific role models, feeling culturally safe, and the effect of the group as a support system. Participants liked the community hall venue and being able to attend sessions outside normal working hours. This compared favourably with the less-welcoming environment of previous clinical spaces they had encountered, with parking and accessibility issues. Participants were happy with the quality and quantity of the information provided during the five group sessions. Feedback indicated that the overall content of the preoperative sessions was consistently positive with a mean score of 4.7/5 across all survey items. All respondents “strongly agreed” with four statements: “I felt safe when raising my point of view in meetings”, “I felt emotionally safe”, “I was made to feel welcome”, and “I left the sessions with useful information”. See Fig. 1 for further details of feedback on the overall content of the information sessions.

**Fig. 2:**
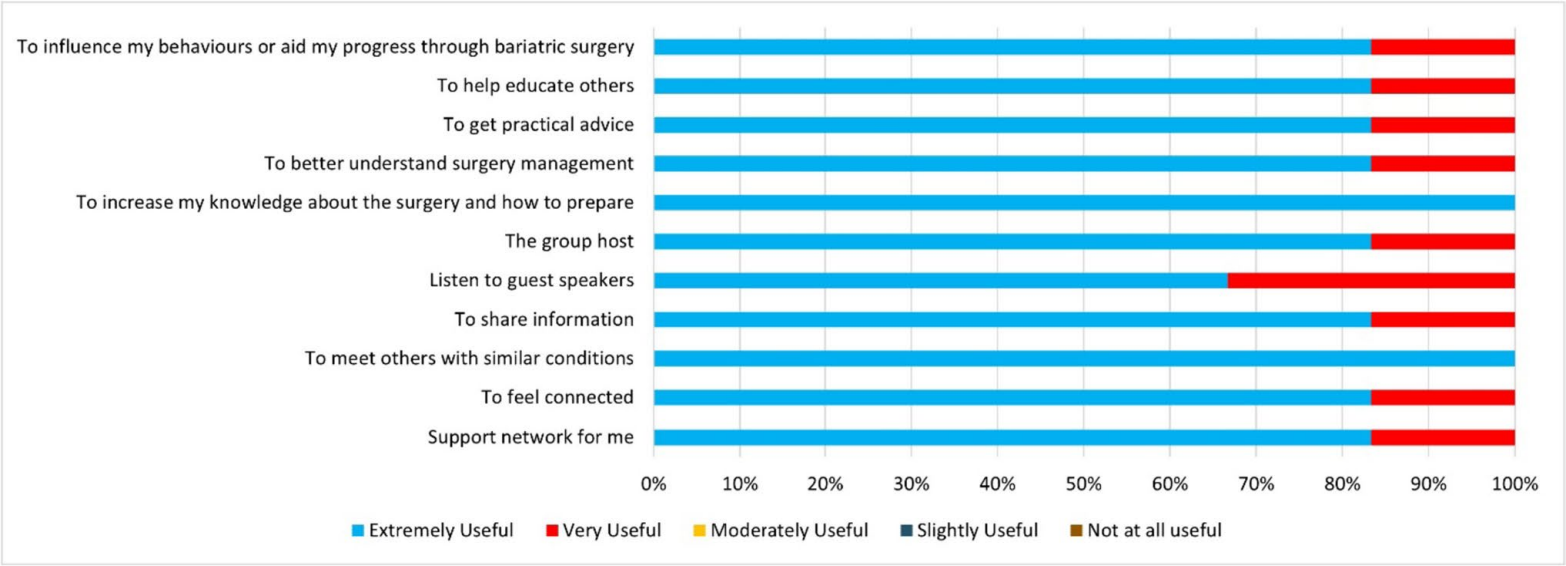
Overall usefulness of the program

Feedback on the program’s overall usefulness was consistently positive, with a mean score of 4.8/5 (see Fig. 2).

## Responses to the Open-Ended Questions

Respondents’ comments indicated that the sessions were conducted in a culturally competent manner: “warm, welcoming, acknowledged our Pasifika-ness and values of inclusiveness, *aroha, manaakitanga* and space”. Some participants felt comfortable asking questions in this environment: “Everything was on point. I felt comfortable in the surroundings to raise my hand if I needed to ask questions”. One participant pointed out that given this was the first prototype of such a program, there would need to be some aspects that would evolve and need to be adjusted: “It’s an evolving process, not the perfect cookie cut, the beginning of many things, and some things may need to be adjusted”. Some participants indicated that the sessions were informationally relevant, and at the same time, there could have been more of a focus on being accountable to yourself. In response to the question, “How hopeful about the surgery process do you feel after attending the group sessions?”, 83% of respondents were “very hopeful”, and the remainder were “somewhat hopeful”. Respondents’ comments highlighted the supportive relationships built among group members during the program. This is reflected in the following comment:

The strength of the sessions has been as much about the support and relationships built with our Pacific cohort as it has been about the speakers that came to our meetings. The collegiality, trust and vulnerability. Without the connection to our group... I don’t know if I would have made it through these last 18 months.

## Future of Program

When asked whether the Pacific-led program should be made available to patients in the future, all respondents answered in the affirmative. One participant indicated that they felt that Pacific patients should have to opt out of the program rather than opt into it. Another participant recognized that the program would lead to higher success rates for Pacific people. One participant felt the program offered a safe space due to being conducted in a Pacific social context:

... No one can walk this journey on their own… It is important that we can have a safe space [where] we can voice our concerns and have like-minded people who are on the same journey support us every step of the way. The Pasifika stream is so vital because our psyche is different. Our social construct is different.

## Discussion

The primary aim of this study—to develop and evaluate a Pacific-led preoperative weight loss surgery program aimed at improving retention—was achieved despite the global pandemic and associated lockdowns. After an initial development and recruitment phase, the formal program ran from October 2020 to July 2021, when the last preoperative session was held. From a research viewpoint, the study continued beyond this point, with the deployment of the post-preoperative session survey and the ongoing recording of video diaries. The retention rate was 43%, which is substantially higher than the mainstream program’s retention rate between 2007 and 2016 of 27% [11]. However, this result must be viewed cautiously because the sample size was too small for this increase to be tested for statistical significance. As such, larger studies and a randomized control trial would be needed to support the current findings.

The components contributing to the success of the current program have also been identified in previous studies. A qualitative interview study exploring patient experiences (*n* = 12) of a bariatric program for managing obesity in Southwest England found that participants felt that the group was a resource for a lifestyle change, there was a sense of self that was based upon their participation in the group, psychological connections to other patients were established, and shared social identity was regarded as a key feature through which the program’s educational material was accessed [17]. Participants’ weight became a “problem” through a collective lens, empowering the initiation of a sustained individual lifestyle change. In the current study, feedback indicates that the collective cohesion of the group was a key factor in supporting participants’ progress.

Additionally, co-designing the program with the surgery team and Pacific role models effectively achieved a culturally anchored format. One recent community-centre diabetes-prevention intervention study co-designed by youth, health providers, and researchers found that co-designing interventions for Pacific populations needed to be culturally tailored to meet the realities of the communities [18]. One of the themes they identified, namely “Building a safe space”, was also achieved in the current study, with participants indicating the importance of journeying with the group and for accountability. Despite not being a formal part of the program, the support group (also known as the *Aiga* or “bari family”) appears to have played a major role in boosting patient satisfaction and preventing attrition. Having grown out of the preoperative sessions as a face-to-face phenomenon, it moved online during the COVID-19 lockdown and persisted well beyond the completion of the program and the study.

A limitation of the study was that due to resourcing constraints, there were no bariatric multi-disciplinary team (MDT) members working on the study who were of Pacific descent. This may have affected retention rates and quality of service and experiences for participants in the study, due to the potential gaps in Pacific cultural competency levels, of the MDT team members. Previous research has found that non-Pacific providers may lack an understanding of Pacific collectivist cultural values and practices [19]. Although one answer to this issue going forward would be to employ Pacific health professionals on MDT teams, there is a significant and ongoing paucity of Pacific health professionals available to work in weight loss surgery MDT roles across NZ [20]. As such, it would not be practical to expect all members of a bariatric MDT taking part in a Pacific-led preoperative program to be of Pacific descent. One way to approach this gap is an intervention aimed at developing the knowledge and Pacific cultural competency [21, 22] of all non-Pacific MDT members. Taking this limitation into account, the current study overall supports the need for affirmative action at the policy level to include Pacific-led preoperative weight loss surgery programs into existing publicly funded weight loss surgery programs.

## Translations

Talanoa: talk or discussion.

Aroha: to love, feel pity, feel concerned for, feel compassion, empathize.

Manaakitanga: hospitality, kindness, generosity, support—the process of showing respect, generosity, and care for others.

## References

[1] Adams TD, Davidson LE, Litwin SE (2017). Weight and metabolic outcomes 12 years after gastric bypass. N Engl J Med.

[2] Cornejo-Pareja I, Molina-Vega M, Gómez-Pérez AM (2021). Factors related to weight loss maintenance in the medium-long term after bariatric surgery: a review. J Clin Med.

[3] English WJ, DeMaria EJ, Hutter MM (2020). American Society for Metabolic and Bariatric Surgery 2018 estimate of metabolic and bariatric procedures performed in the United States. Surg Obes Relat Dis.

[4] Sundström J, Bruze G, Ottosson J (2017). Weight loss and heart failure: a nationwide study of gastric bypass surgery versus intensive lifestyle treatment. Circulation.

[5] Müller TD, Blüher M, Tschöp MH (2022). Anti-obesity drug discovery: advances and challenges. Nat Rev Drug Discov.

[6] De Lorenzo A, Romano L, Di Renzo L (2020). Obesity: a preventable, treatable, but relapsing disease. Nutrition.

[7] Rubino F, Schauer PR, Kaplan LM (2010). Metabolic surgery to treat type 2 diabetes: clinical outcomes and mechanisms of action. Annu Rev Med.

[8] Ministry for Pacific Peoples. Pacific Aotearoa status report: a snapshot. 2020. Retrieved from https://www.mpp.govt.nz/assets/Reports/Pacific-Peoples-in-Aotearoa-Report.pdf.

[9] Rahiri JL, Coomarasamy C, MacCormick A (2020). Ethnic disparities in access to publicly funded bariatric surgery in South Auckland New Zealand. Obes Surg.

[10] Garrett M, Poppe K, Wooding A (2020). Private and public bariatric surgery trends in New Zealand 2004–2017: demographics, cardiovascular comorbidity and procedure selection. Obes Surg.

[11] Taylor T, Wang Y, Rogerson W (2018). Attrition after acceptance onto a publicly funded bariatric surgery program. Obes Surg.

[12] Rahiri JL, Lauti M, Harwood M (2018). Ethnic disparities in rates of publicly funded bariatric surgery in New Zealand (2009–2014). ANZ J Surg.

[13] Taylor T, Wrapson W, Dewes O (2019). Preoperative bariatric surgery programme barriers facing Pacific patients in Auckland, New Zealand as perceived by health sector professionals: a qualitative study. BMJ Open.

[14] Taylor T, Wrapson W, Dewes O et al. Fear, stigma and experience: Pacific patients weight loss surgery perspectives. [Manuscript in preparation]. Department of Molecular Medicine, University of Auckland. 2024.

[15] Pulotu-Endemann, F. K. Fonofale: Model of health. 2001. Retrieved December 2023, from: https://hpfnz.org.nz/assets/Fonofalemodelexplanation.pdf.

[16] Vaioleti TM (2006). Talanoa research methodology: a developing position on pacific research. Waikato J Ed.

[17] Tarrant M, Khan SS, Farrow CV (2017). Patient experiences of a bariatric group programme for managing obesity: a qualitative interview study. Br J Health Psychol.

[18] Prapaveissis D, Henry A, Okiama E (2022). Assessing youth empowerment and co-design to advance Pasifika health: a qualitative research study in New Zealand. Aust N Z J Public Health.

[19] Fa’alogo-Lilo C, Cartwright C (2021). Barriers and supports experienced by Pacific peoples in Aotearoa New Zealand’s mental health services. J Cross Cult Psychol.

[20] Pacific Perspectives. January 2013. Pacific Health Workforce Service Forecast. Wellington: Pacific Perspectives. Retrieved in December 2023, from https://www.health.govt.nz/publication/pacific-health-workforce-service-forecast.

[21] Tiatia-Seath J (2018). The importance of Pacific cultural competency in healthcare. Pac health dialog.

[22] Tiatia J. 2008. Pacific Cultural Competencies: A literature review. Wellington: Ministry of Health. Retrieved in December 2023, from https://www.health.govt.nz/system/files/documents/publications/pacific-cultural-competencies-may08-2.pdf.

